# A Risk-Based Isolation Strategy for MDR-Endemic Facilities with Limited Resources

**DOI:** 10.3390/idr18030044

**Published:** 2026-05-09

**Authors:** Zeynep Ture, Emine Alp

**Affiliations:** 1 Department of Infectious Diseases, Gürlife Hospital, Eskişehir 26320, Türkiye; 2 Department of Infectious Diseases and Clinical Microbiology, Faculty of Medicine, Ankara Yıldırım Beyazıt University, Ankara 06050, Türkiye; 3 Department of Infectious Diseases and Clinical Microbiology, Ankara Bilkent City Hospital, Ankara 06800, Türkiye

**Keywords:** multidrug resistance, contact isolation, Risk Classification, infection control, VRE, ESBL, CRE

## Abstract

Background/Objectives: The increasing burden of multidrug-resistant (MDR) microorganisms and limited resources in healthcare settings are making traditional strategies based on routine isolation of all carriers unsustainable. Methods: A clinical narrative review was conducted by searching PubMed, Web of Science, and Google Scholar for studies published between 2011 and 2025. International guidelines were analyzed to synthesize a sustainable infection control strategy. Results: High-quality evidence, including cluster-randomized trials, indicates that routine contact isolation for endemic ESBL-producing Enterobacterales (IRR: 0.99) and VRE (RR: 0.93) provides no additional benefit over standard precautions. In contrast, strict isolation remains vital for high-threat pathogens such as Carbapenem-Resistant Enterobacterales (CRE), *Acinetobacter baumannii* (CRAB), and *Candidozyma auris* due to their high environmental resilience and limited treatment options. Prioritization should be guided by pathogen biology, patient-specific transmission traits (e.g., diarrhea), and facility infrastructure. Conclusions: Traditional one-size-fits-all infection control is increasingly unsustainable under resource constraints. A risk-based approach prioritizing horizontal measures for low-risk pathogens enables a more balanced allocation of limited resources toward high-threat containment.

## 1. Introduction

Antimicrobial resistance is described as a silent pandemic for global public health and is projected to cause 10 million deaths annually by 2050 [1]. Vertical measures involve active surveillance cultures targeting specific pathogens and the implementation of strict contact precautions (use of private rooms, gowns, and gloves) for infected or colonized patients [2]. Notably, the 2014 ESCMID guidelines strongly recommended the implementation of contact precautions even in endemic settings [2]. However, as of 2025, infection control professionals and healthcare administrators face a critical paradox. While the prevalence of resistant pathogens is increasing, the physical and human resources required to isolate these patients are being exhausted [3]. Attempting to implement universal isolation in highly endemic regions leads to systemic overload, as the number of colonized patients often exceeds the available single-room capacity and staffing ratios [3]. The search and destroy strategy, which is successful in low-prevalence countries like the Netherlands, is mathematically and logistically inapplicable in highly endemic regions such as Turkey, Greece, and Italy. A six-center European study conducted by Van Dijk et al. (2022) demonstrated that hospitals with the highest prevalence of MDR organisms paradoxically applied the fewest screening and isolation measures [4].

This resource scarcity has begun to transform practical hospital applications [5]. According to data from the SHEA Research Network, 35% of U.S. hospitals have completely discontinued routine contact precautions for Methicillin-resistant *Staphylococcus aureus* (MRSA) and Vancomycin-resistant *Enterococcus* (VRE); this rate was reported as only 7% in 2015 [6]. This shift reflects a growing clinical consensus that horizontal measures provide comparable safety with significantly lower resource consumption.

While previous literature has extensively covered vertical and horizontal strategies, a significant gap remains in providing a practical, resource-optimized prioritization model for facilities where universal isolation is logistically impossible. This review fills this gap by introducing the Precision Infection Control framework.

## 2. Materials and Methods

This review was clinical narrative in nature, yet it employed a structured and transparent search strategy to ensure methodological robustness and reproducibility. A comprehensive literature search was conducted in the PubMed, Web of Science, and Google Scholar databases for peer-reviewed articles published between January 2011 and December 2025. The search employed specific keywords including ‘multidrug-resistant organisms,’ ‘contact isolation,’ ‘de-escalation,’ ‘horizontal measures,’ and ‘risk-based prioritization’.

**Figure 1:**
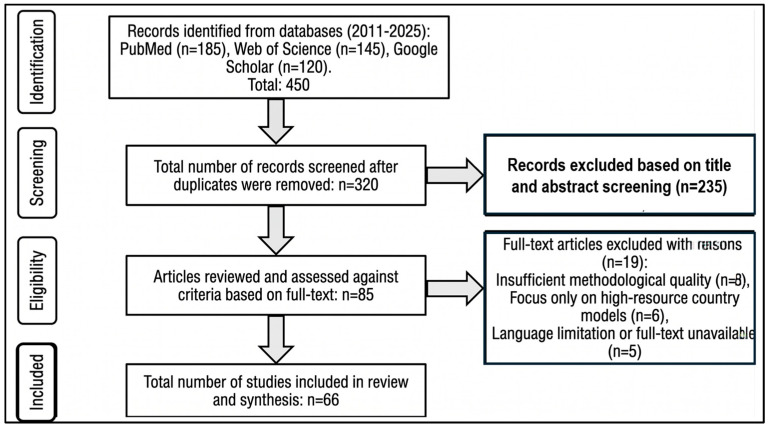
Flowchart of the Study Selection Process.

To synthesize a sustainable infection control model, inclusion criteria focused on high-impact original research, meta-analyses, and current clinical guidelines from international health organizations, including the World Health Organization (WHO), Centers for Disease Control and Prevention (CDC), and European Society of Clinical Microbiology and Infectious Diseases (ESCMID). Studies were screened based on their relevance to MDR-endemic and resource-limited healthcare settings. To enhance transparency, a PRISMA-style filtering logic was applied to the selection process. Initial records were screened by title and abstract, followed by a full-text review to evaluate methodological quality and the strength of evidence. Higher priority was given to Level 1 evidence, such as randomized controlled trials and systematic reviews. Although not a formal systematic review, we adopted PRISMA-informed methods to enhance transparency and robustness in our literature identification process (Figure 1).

**Artificial Intelligence Statement:** During the preparation of this manuscript, the authors utilized Google Gemini (Version 5.0, Google LLC, Mountain View, CA, USA) for language editing and structural refinement of the text. After using this tool, the authors reviewed and edited the content as needed and take full responsibility for the final version and the clinical accuracy of the publication.

## 3. Evidence Synthesis: Clinical Impact of Contact Isolation

The traditional approach is that contact isolation reduces transmission. However, high-quality studies in recent years have called this tradition into question, especially for endemic Gram-negative bacteria and VRE (Table 1).

### 3.1. Extended-Spectrum Beta-Lactamase-Producing Enterobacterales: Is Isolation Necessary?

The transmission dynamics of ESBL-producing *Escherichia coli* and *Klebsiella pneumoniae* within the hospital environment differ fundamentally from those of MRSA [13]. The R-GNOSIS study group provided robust evidence by analyzing over 38,000 admissions. Their findings showed no significant difference in healthcare-associated ESBL incidence between periods of strict contact isolation and standard precautions (Level-1) [7]. This finding is further corroborated by the systematic review by Prevel et al. (2019) reviewing 25 studies conducted in Intensive Care Units (ICUs) and revealed that systematic screening for ESBL-E carriage combined with contact precautions failed to reduce cross-transmission rates (Level-1) [14]. This real-world data reinforces the conclusions drawn from controlled trials [15]. In a study conducted by Thompson et al., the infection rate did not increase after the end of hospital-wide isolation; on the contrary, the incidence per 10,000 patient-days decreased from 3.71 to 3.00 (Level-2) [16]. Musumeci et al. reported no increase in the incidence of hospital-acquired ESBL during a 5-year follow-up after the end of contact tracing measures in Switzerland (Level-2) [17].

Consequently, current high-level evidence suggests that routine contact isolation may not provide a significant additional benefit over standard precautions for ESBL-E in endemic settings; however, this should be interpreted cautiously within the context of the study’s specific epidemiological setting and existing horizontal measures ) [18].

Comparison of vertical and horizontal approaches in infection control is shown in Table 2.

### 3.2. VRE: Management Without Isolation and Genomic Evidence

Vancomycin-Resistant Enterococci (VRE) continues to be a significant cause of healthcare-associated infections; however, traditional isolation policies regarding the management of this pathogen are increasingly being questioned. A recent meta-analysis evaluated the safety of discontinuing private room or cohort isolation for VRE patients in endemic or non-outbreak settings. The results of the analysis showed that the discontinuation of isolation did not cause a significant increase in hospital-acquired VRE infection rates or VRE bacteremia (Level-1) [8]. This finding emphasizes that the discontinuation of isolation may be safe, provided it is supported by horizontal measures such as hand hygiene compliance >84%, daily chlorhexidine bathing, and rigorous environmental cleaning (Level-1) [8]. This epidemiological data is also supported by genomic evidence [9]. This study demonstrates that isolation alone is not a solution, and the primary safeguard is horizontal measures that reduce the bacterial load. However, the data regarding the discontinuation of isolation is not always so clear. An interrupted time-series analysis conducted in South Korea reported that removing the requirement for single-room isolation did not change VRE bacteremia rates, but there was a trend toward an increase in the rate of new VRE colonization. However, the clinical significance of increased colonization should not be underestimated. Evidence suggests a strong correlation between VRE colonization and the subsequent risk of bloodstream infections (BSI), particularly in vulnerable populations. A meta-analysis involving patients with malignancies demonstrated that VRE colonization significantly increases the risk of VRE-BSI, emphasizing that colonization often serves as a precursor to life-threatening sepsis. Furthermore, VRE colonization has been associated with increased mortality in septic patients, highlighting the need for caution when de-escalating isolation measures in high-risk units (Level-1) [19,20,21]. However, a critical observation in their findings was a discernible trend toward increased VRE colonization among patients. This highlight suggests that while horizontal measures effectively prevent invasive infections, they may be less potent than contact precautions in controlling the overall environmental bioburden and subclinical transmission of VRE [10].

A study comparing the period of routine screening to the period when screening was performed only upon suspicion of an outbreak, no significant increase was found in the incidence of healthcare-associated VRE bacteremia. Although a slight increase in colonization rates was observed following the cessation of screening, this situation did not translate into clinical infections (Level-2) [22]. This finding is also consistent with the study by Ulu-Kılıç et al., which included 6372 pediatric patients. In this study, 239 patients (3.75%) were found to be colonized, but VRE infection developed in only 3 patients (0.04%). While the annual cost of screening all patients reached approximately 19,000 dollars (Level-2) [23]. In contrast, a modeling study conducted in Canada suggested that screening and isolating high-risk patients in general internal medicine wards could be cost-effective in the long term by preventing infections [24].

In conclusion, discontinuation of isolation should not be considered safe/routine in all endemic settings; rather, it is specified as an alternative practice only applicable where prevalence is low and compliance with strong hand hygiene and antiseptic bathing practices is high. Therefore, maintaining targeted screening and isolation in high-risk units, such as pediatric oncology or ICUs, remains the most balanced approach in terms of patient safety, regardless of resource limitations.

### 3.3. MRSA: Transition from Vertical Measures to Horizontal Measures

Methicillin-resistant *Staphylococcus aureus* has been the pathogen for which the search and destroy strategy has been implemented most strictly and for the longest duration in healthcare-associated infections. However, recently published data indicate that the policy of routine active surveillance and contact isolation for MRSA has failed to demonstrate the expected effect in endemic situations. A systematic review and meta-analysis conducted by Marra et al., revealed that the discontinuation of contact precautions did not lead to a statistically significant increase in MRSA infection rates (Level-1) [25]. A large-scale retrospective study reported from an 800-bed research hospital also stated that the discontinuation of contact precautions did not lead to any increase in healthcare-associated MRSA bacteremia or surgical site infections (Level-3) [26]. Despite these findings favoring de-escalation, recent epidemiological shifts necessitate a more cautious evaluation. According to 2024/25 data from the UK Health Security Agency (UKHSA), MRSA bloodstream infections in England reached a 14-year high with 1064 reported cases—an approximately 30% increase compared to 2018/19 levels. This resurgence raises concerns that discontinuing contact precautions may be premature in regions experiencing upward trends in MRSA incidence (Level-1) [27].

These epidemiological data are also supported by recent evidence from the pediatric population. A study conducted in a Pediatric ICU demonstrated that discontinuing contact isolation for patients colonized or infected with MRSA and VRE resulted in a significant reduction in personal protective equipment costs (Level-2) [28]. In the retrospective study by Most et al. was found that universal MRSA screening at hospital admission did not reduce the rates of healthcare-associated MRSA bacteremia compared to no screening (Level-3) [29].

A study in Saudi Arabia reported that discontinuing contact precautions did not increase MRSA rates but rather contributed positively to bed management and staff satisfaction [30]. In conclusion, while a transition to horizontal measures is supported in stable endemic settings, the management of MRSA must remain adaptive to local epidemiological data. In facilities or regions witnessing a resurgence, as seen in recent UK reports, maintaining current levels of contact isolation and active screening remains a vital safeguard against hospital-acquired transmission.

### 3.4. Carbapenem-Resistant Enterobacterales (CRE), Carbapenem-Resistant Acinetobacter baumannii (CRAB), and the Necessity of Isolation

In contrast to ESBL, the management of Carbapenem-Resistant Enterobacterales (CRE) remains critical, as prospective data indicate a high ICU acquisition rate of 8.5%, predominantly through asymptomatic colonization. The arguments presented for relaxing isolation measures regarding ESBL, VRE, and MRSA lose their validity when it comes to Carbapenem-Resistant Enterobacterales and Carbapenem-Resistant *Acinetobacter baumannii* (CRAB) [31]. Unlike ESBL-positive *E. coli,* these pathogens carry a high transmission potential due to their ability to survive for long periods on surfaces in the hospital environment and their capacity to form biofilms [32]. A prospective study by Wu et al. demonstrated that 67.6% of CRE acquisitions in ICUs occur as asymptomatic colonization (Level-2) [12]. The study by Ben-David et al. emphasizes that a recurrence rate of 26.6% is observed within the first year even in patients thought to have cleared their carriage; therefore, early discontinuation of isolation may be risky [33]. Given these risks, it is recommended that patients colonized with this group of bacteria be kept in strict contact isolation in single rooms if possible [34]. Consequently, the strategy of transitioning to standard precautions proposed for ESBL in the Precision Infection Control model is not an option for CRE and CRAB; on the contrary, limited resources should be channeled toward active surveillance and strict isolation of this group.

### 3.5. Candidozyma auris: Environmental Persistence and the Necessity of Isolation

Despite increasing evidence for the relaxation of contact precautions in the management of endemic MDR microorganisms this approach is strictly inapplicable to *Candidozyma auris.* The CDC and numerous national guidelines recommend that all patients detected with *C. auris*, regardless of risk factors, be placed in contact isolation in single rooms and that isolation be maintained until discharge (Level-1) [35,36,37,38]. A genomic analysis of an outbreak that occurred in an ICU between 2021 and 2023 revealed that patient rooms could become re-contaminated only 4 h after terminal cleaning and disinfection. More importantly, *C. auris* is resistant to standard hospital disinfectants (particularly quaternary ammonium compounds). For this reason, guidelines mandate the use of chlorine-based disinfectants (e.g., 1000 ppm sodium hypochlorite) or hydrogen peroxide-based products even in routine cleaning (Level-1) [35,36,37,38,39].

*C. auris* colonization can rapidly progress to invasive infections, especially in ICU patients [40]. A 4-year cohort study reported that 14.2% of candidemia in the ICU were caused by *C. auris,* and fluconazole resistance in these patients reached 94.7% [41]. Advanced age, the presence of central venous catheters, and the use of broad-spectrum antibiotics emerge as independent risk factors for the development of infection (Level-1) [35,36,37,38]. Since *C. auris* can re-contaminate patient environments as quickly as 4 h after cleaning and exhibits intrinsic resistance to common quaternary ammonium-based disinfectants, standard manual cleaning protocols are often insufficient. Advanced environmental decontamination technologies, such as Hydrogen Peroxide Vapor and Ultraviolet-C light disinfection, offer superior efficacy in eradicating the environmental bioburden. These ‘no-touch’ automated systems should be considered as essential adjuncts to routine chlorine-based disinfection to prevent persistent outbreaks and cross-transmission in high-risk units [35,36,37,38].

Unlike MRSA, there is no effective decolonization protocol for *C. auris*, and routine decolonization is not recommended. Therefore, removing contact precautions for *C. auris* carriers is not safe. On the contrary, resources should be channeled toward active surveillance of this patient group and strict contact (Level-1) [35,36,37,38].

## 4. Ethical, Economic, and Psychological Dimensions of Contact Isolation Practices

### 4.1. Ethical Perspective and the Principle of Proportionality

Infection control measures must be based on the proportionality principle of public health ethics [42]. Isolating an asymptomatic patient for months despite a low risk of transmission can be considered an unethical practice that restricts the patient’s freedom and diminishes the quality of care. In addition, Rump et al. (2020) takes this issue a step further by proposing that MDR microorganisms’ management be reframed as a matter of solidarity [42]. While countless undetected carriers circulate freely in the community, restricting only those who admit to the hospital with strict isolation measures creates an ethically arbitrary inequality and injustice [42]. Isolation must be seen as a last resort, adhering to the principle of proportionality to prevent arbitrary inequality among patients.

### 4.2. Patient Experience: Loneliness and Stigmatization

**Figure 2:**
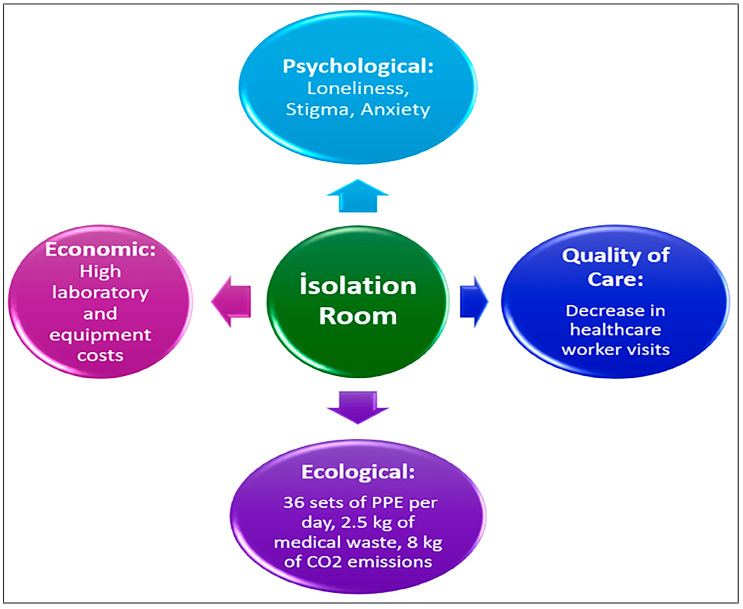
The Multi-dimensional Hidden Costs of Routine Contact Isolation.

The qualitative study conducted by Farrukh et al. (2025) strikingly reveals the experiences of patients in isolation. Patients report feeling lonely and stigmatized; they state that healthcare workers’ entries into the room have decreased and that communication has broken down [43]. There are studies reporting that isolation is an independent risk factor that increases hospital anxiety and depression [44]. The multi-dimensional hidden costs of routine contact isolation are shown in Figure 2.

Data-driven metrics highlight a substantial daily ecological impact, including 36 sets of personal protective equipment, 2.5 kg of hazardous medical waste, and 8 kg of CO_2_ emissions per patient-day in isolation. These figures underscore the hidden operational burden that necessitates a transition to more sustainable, risk-based isolation strategies [45,46].

### 4.3. Economic Burden and Resource Management

Specifically in developing countries, screening and isolating everyone’s strategy leads to financial difficulties. A study conducted at a tertiary university hospital in a developing country reported that the annual laboratory cost of screening all patients in the pediatric population reached 19,000 dollars, yet the detected infection rate was only 0.04% [21,22,23]. This low rate demonstrates the necessity of targeted strategies instead of universal screening [24]. From a policy perspective, the transition toward ‘Precision IPC’ is not merely a clinical preference but a strategic necessity that aligns with global sustainable healthcare frameworks, such as the WHO Global Antimicrobial Resistance surveillance goals. By conducting a quantitative cost–benefit analysis contrasting the high annual screening costs of $19,000 against a minimal 0.04% infection detection rate. It becomes evident that current universal isolation models are economically inefficient. This paradigm shift supports a more resilient healthcare system by redirecting limited financial resources toward high-impact containment of epidemic-prone threats [23,35].

## 5. Risk-Based Prioritization

An often overlooked but critically important dimension of isolation measures in the era of the global climate crisis is environmental sustainability. The healthcare sector is responsible for approximately 4.4% of global net carbon emissions, and a large portion of this ratio consists of single-use plastic materials [45,46]. Unlike traditional IPC frameworks that often treat all MDR carriers as equal threats or rely solely on basic hygiene (horizontal), the Precision IPC model offers a multi-layered risk assessment, ensuring that intensive resources are reserved for the highest-threat scenarios [47].

**Figure 3:**
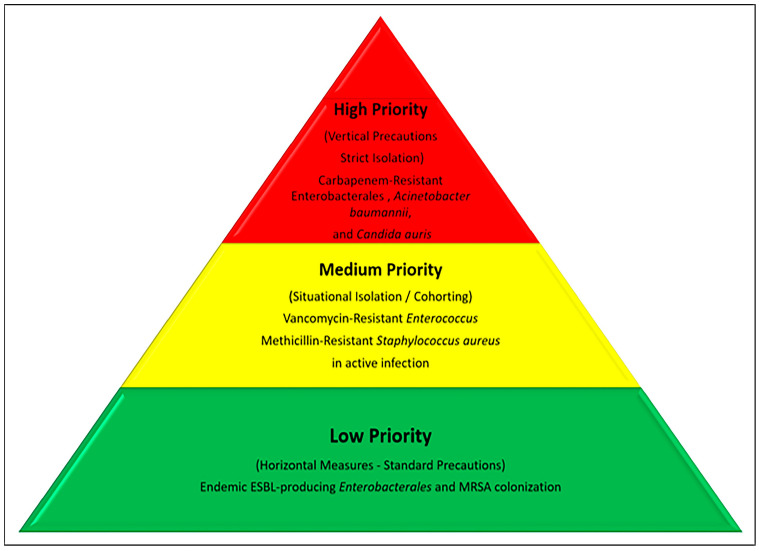
Risk-Based Isolation Priority Pyramid.

In the present day, the isolation of every patient approach must give way to isolating the right patient at the right time approach. The model developed by Alp Meşe et al. (2025) categorizes isolation decisions into three risk categories based on Patient, Pathogen, and Healthcare Facility factors (Figure 3) [47].

The mechanistic linkage between the tiers of the Priority Pyramid is defined by the synergistic risk of pathogen virulence, host vulnerability, and environmental stability. To translate this conceptual model into clinical action, we propose a three-step decision algorithm:

1. Pathogen Screening: Identify the microorganism’s environmental resilience (>1 month vs. <1 day) and mortality risk.

2. Patient Profiling: Assess the presence of ‘super-spreader’ traits, such as diarrhea, incontinence, or multiple colonized sites, alongside the patient’s immune status.

3. Infrastructure Audit: Evaluate current single-room occupancy and nurse-to-patient ratios to determine if cohorting or standard precautions are the only viable alternatives.

### 5.1. Patient-Related Risk Factors

The first step in the isolation decision is the assessment of the patient’s transmission potential and susceptibility to infection. While traditional IPC models, such as the 2014 ESCMID guidelines, strongly favored vertical measures regardless of the setting, they often failed to account for the systemic overload in MDR-endemic regions. In contrast to the rigid vertical vs. horizontal dichotomy, the Precision IPC model is inherently adaptive. It allows for the de-escalation of resources from low-threat, low-resilience pathogens like ESBL-producing *E. coli* to high-priority threats such as CRE and *C. auris*, thereby offering a more sustainable and generalizable framework for facilities with limited resources.

#### 5.1.1. Symptomatic Infections and Immunosuppression (High Priority)

Patients with symptomatic infections shed significantly more bacteria into the environment compared to asymptomatic carriers. Therefore, symptomatic patients are indisputably in the high priority group and should be isolated in a single room [47]. Immunosuppressed patients are at higher risk. They experience more severe infections and have a higher likelihood of progressing from colonization to active infection [48]. Almohaya et al. (2024) demonstrated that MDR colonization increases mortality by 3.94 times in SOT recipients. These patients should be evaluated in the absolute isolation category, even in cases of resource limitation [11].

#### 5.1.2. Super-Spreader Characteristics (High Priority)

Not every carrier poses an equal risk. Studies demonstrate that only 18% of carriers are responsible for 80% of environmental contamination in the hospital setting [49]. Patients with fecal incontinence, diarrhea, or colonization in multiple body sites shed bacteria intensely into the environment. These patients, even if asymptomatic, should be considered super-spreaders and prioritized for isolation [47,50].

#### 5.1.3. Intensity of Medical Intervention and ICU Length of Stay (Medium Priority)

Patients requiring intensive medical intervention with numerous invasive devices can serve as a source of transmission and are also highly susceptible to infection themselves. Wu et al. (2023) found that the risk of CRE acquisition increased 5.49-fold in patients staying in the ICU for more than 3 weeks [12]. Therefore, patients for whom a prolonged ICU stay is anticipated are in the “Medium Priority” group; if a single room is unavailable, cohorting should be implemented.

#### 5.1.4. Asymptomatic Colonization and Short Length of Stay (Low Priority)

This is the group where the discontinuation of isolation is safest. Patients with asymptomatic colonization who have no invasive devices, are fully continent, and do not have diarrhea are in the low priority group. Particularly for carriers of ESBL-producing *E. coli*, since the risk of intra-hospital transmission has been proven to be very low, it has been argued that standard precautions are sufficient instead of isolation [51,52].

### 5.2. Pathogen-Related Risk Factors

The isolation decision depends not only on the patient but also on the biology of the microorganism they carry [53].

#### 5.2.1. Mode of Transmission

Pathogens that can be transmitted through the air or spread via droplets spread much faster and are more difficult to control than those transmitted by contact. Alp Meşe et al. state that pathogens transmitted via the air are high priority, whereas those transmitted only by contact should be evaluated according to their risk level [47].

#### 5.2.2. Transmissibility Coefficient (R0 Value)

Pathogens with a reproduction number (R0) > 2.5 (high infectivity) are in the high priority group due to their outbreak potential. In contrast, for pathogens with a low R0 value (<2.5) that are already widespread in the community, isolation measures can be shifted to “Medium” or “Low” priority [47].

#### 5.2.3. Environmental Resilience and Persistence

How long a microorganism remains viable on inanimate surfaces is critical for the isolation decision.

*High Resilience (>1 Month): Acinetobacter baumannii*, *C. auris*, *Clostridioides difficile* (spores), and VRE. These pathogens can survive for weeks even on dried surfaces. Since environmental cleaning is difficult, patients carrying these pathogens should be isolated with “High Priority”.

*Low Resilience (<1 Day):* Some Gram-negatives, such as ESBL-producing *E. coli,* cannot survive long on dry surfaces. Therefore, the risk of environmental transmission is low, and these patients can be placed in the low priority group.

#### 5.2.4. Mortality Rate and Treatment Options

The risk of the infection caused by the pathogen being fatal and the available antibiotic options are also criteria. Pathogens with no treatment options, such as *C. auris* or Pan-drug-resistant bacteria, are agents whose spread must be halted as quickly as possible [47,53].

### 5.3. Healthcare Facility-Related Risk Factors

This is the point where the gap between the ideal world and the real world widens. The isolation strategy must be adapted according to the institution’s capacity.

#### 5.3.1. Infrastructural Inadequacy and Isolation Room Capacity

If the ratio of single rooms in a hospital is <10%, it is mathematically impossible to isolate everyone [4]. In this case, the inadequate infrastructure factor comes into play, and the institution must allocate isolation rooms only to the High Priority group [47,54].

#### 5.3.2. Infection Control Program and Staffing Workload

If the Infection Control Program in an institution is inadequate, if the number of personnel is low (low nurse-to-patient ratio), or if compliance with measures is low, the risk of transmission increases. In hospitals with low compliance or a low staffing ratio, the risk must be perceived as higher, and isolation measures must be kept stricter [47].

#### 5.3.3. Financial Support and Surveillance Capacity

Hospitals that have a regular surveillance program and the availability of rapid diagnostic tests can safely remove low-risk patients from isolation because they can detect a problem immediately if one occurs [55]. However, hospitals with insufficient financial support that cannot conduct surveillance may have to act more cautiously (Medium Priority) against the invisible threat [56,57]. Risk-based isolation prioritization is shown in Table 3.

## 6. Implementation Strategies and Proposed Solutions

### 6.1. Discontinuing Isolation: Active Follow-Up

The traditional once positive, always positive approach leads to bed management crises and unnecessary resource consumption, particularly in endemic regions. The decision to discontinue isolation should be made by considering the patient’s clinical status, the biology of the pathogen, and the institution’s level of endemicity (Table 4).

#### 6.1.1. ESBL and VRE

Relaxed Criteria and Clinical Judgment For ESBL-producing Enterobacterales and VRE, the Society for Healthcare Epidemiology of America guidelines support the removal of contact precautions in endemic situations [58]. Traditionally, discontinuation of isolation for ESBL and VRE required three consecutive negative rectal swabs. However, this resource-intensive approach is increasingly challenged. Large-scale evidence, including the STAR*ICU trial and the CDC Prevention Epicenters Network studies [59,60]. has demonstrated that such intensive screening strategies do not offer superior outcomes compared to robust horizontal measures like hand hygiene and environmental cleaning [60]. For these low-risk pathogens, requiring serial negative tests shifts critical resources toward the microbiology laboratory without clear clinical benefit. Therefore, de-isolation for ESBL and VRE should be based on clinical stability and adherence to horizontal precautions rather than routine re-screening. This shift aligns with the findings of Bearman et al., who emphasize that VRE colonization is prolonged and screening costs are high, suggesting that isolation can be discontinued without re-sampling in patients who have remained clinically stable for a specific duration, such as 3–6 months [61].

In low-risk units, a time-based de-isolation approach (removing isolation without screening for patients whose last positive culture was over 6 months ago and who have shown no signs of infection during this period) may be a rational strategy for resource management.

#### 6.1.2. MRSA

CDC and UK Health Security Agency guidelines recommend three negative screening results obtained at least 24 h (preferably 1 week) apart from a patient not receiving systemic antibiotic therapy to terminate isolation [58,62]. Larsson et al. state that recurrence is observed within one year in 20% of patients who had previously tested negative [63]. Therefore, screening patients whose MRSA isolation was terminated via preemptive isolation during readmission is a safe strategy.

#### 6.1.3. CRE and CRAB

Carbapenem-resistant organisms constitute the most challenging group because they can remain silent in the intestinal flora for long periods and re-emerge under antibiotic pressure. Ben-David et al. (2020) detected recurrence within the first year in 26% of patients thought to have cleared their CRE carriage [30,33]. Consequently, a more cautious approach is necessary; to terminate CRE isolation, a case-based evaluation is required, and obtaining at least 2 consecutive negative rectal swabs taken at least 1 week apart is considered sufficient [64,65].

#### 6.1.4. *Candidozyma auris*

*C. auris* is the most difficult pathogen to eradicate due to the biofilm layers it forms on the skin and in the hospital environment. The CDC recommends that patients colonized with *C. auris* be considered colonized indefinitely. Even if two consecutive negative results are obtained, the risk of patients testing positive again is very high. Therefore, current guidelines do not routinely recommend the discontinuation of isolation for *C. auris* patients [12,32,33,34,35,36,37,38].

### 6.2. Strengthening Horizontal Measures

If isolation is to be discontinued, horizontal measures should be maximized to avoid creating a security vulnerability [66]. Eichel et al. demonstrated that applying daily antiseptic bathing to all ICU patients while removing contact precautions was a critical factor in preventing VRE transmission [9].

### 6.3. Implementation of Metrics and Translational Value

To enhance the translational value of the Precision IPC model, facilities should monitor specific implementation metrics. Real-world data demonstrate that de-escalating isolation for low-risk pathogens can lead to a 20–30% reduction in unnecessary isolation days, directly alleviating bed management crises [33]. Furthermore, evidence from pediatric settings shows that transitioning to horizontal measures for MRSA and VRE results in significant reductions in PPE-related operational costs, without compromising patient safety [28]. These metrics provide a clear benchmark for hospital administrators to evaluate the success of resource reallocation from low-yield screening to the containment of high-threat pathogens like *C. auris* [23].

## 7. Conclusions

In conclusion, the traditional one-size-fits-all approach to contact isolation faces significant sustainability challenges in healthcare facilities operating in MDR endemic settings with limited resources. This review proposes a transition toward a Precision Infection Control model, where resources could be dynamically prioritized based on pathogen virulence, patient-specific traits, and local epidemiology. However, it is important to acknowledge that isolation decisions remain highly context-dependent, and de-escalation strategies must account for the substantial heterogeneity in facility infrastructure and patient risk profiles.

This review has several limitations that warrant consideration. As a narrative review rather than a formal systematic review, it lacks a quantitative synthesis or meta-analytical pooling of risk data. The evidence base is characterized by significant heterogeneity in study designs and settings, which may limit the universal applicability of some conclusions. Furthermore, while the Precision IPC model offers a logical framework for resource reallocation, its long-term safety and efficacy must be formally validated through prospective, multi-center clinical trials before wide-scale implementation.

The Precision Infection Control model represents a shift toward a more sustainable and evidence-based allocation of healthcare resources in MDR-endemic settings. While the Precision IPC framework offers a logical reallocation of resources, its safety and efficacy must be formally validated in prospective, large-scale clinical trials before wide-scale adoption. Ultimately, integrating this risk-stratified approach may be an important component in maintaining high standards of patient safety in an era of increasing antimicrobial resistance.

The implementation of AI-driven surveillance systems and predictive modeling offers the potential to identify ‘super-spreaders’ and high-risk transmission clusters in real-time. By leveraging machine learning algorithms to analyze electronic health records, facilities can move from reactive isolation to a proactive, individualized IPC strategy. Digital surveillance and automated audit tools will be essential to ensure that horizontal measures remain robust while resources are dynamically shifted to meet emerging epidemiological threats.

## Figures and Tables

**Table 1:** Summary of Current Studies Evaluating the Efficacy of Routine Contact Isolation.

Study (Year, Country)	Pathogen	Design	Key Finding	Conclusion	Level of Evidence *
Maechler et al. (2020) [7], Europe	ESBL	Cluster-Randomized (RCT)	Isolation provided no additional benefit to standard precautions (IRR: 0.99)	Discontinuing isolation is safe	1b
Pan et al. (2024) [8], Global	VRE	Meta-analysis	Discontinuation of isolation did not increase VRE infections (RR: 0.93)	Discontinuing isolation is safe	1a
Eichel et al. (2022) [9], Germany	VRE	Time Series	Isolation removed, antiseptic bathing added. Genetic transmission did not increase	Safe when supported by horizontal measures	2b
Kim et al. (2025) [10], Korea	VRE	Time Series	Bacteremia did not increase, but colonization showed an upward trend	Caution is warranted	2b
Almohaya et al. (2024) [11], Global	MDR	Meta-analysis	MDR colonization increases mortality 3.94-fold in SOT recipients	Isolation is MANDATORY	1a
Wu et al. (2023) [12], China	CRE	Prospective	Post-admission acquisition rate in ICU was 8.5%.	Periodic screening is necessary	2b

* Table 1 highlights the heterogeneity across study designs, ranging from high-level evidence (Level 1a meta-analyses) to real-world observational data (Level 2b time-series), providing a comprehensive overview of the current evidence base.

**Table 2:** Comparison of Vertical and Horizontal Approaches in Infection Control.

Feature	Vertical (Pathogen-Specific) Approach	Horizontal (Universal) Approach
Primary Focus	Targeted containment of specific high-risk pathogens (e.g., CRE, CRAB, MRSA)	System-wide reduction in transmission risk for all patients and pathogens
Core Interventions	Active surveillance cultures, contact precautions, cohorting, and preemptive isolation	Universal hand hygiene, chlorhexidine bathing, environmental cleaning, and antibiotic stewardship
Cost	High: Significant investment in laboratory screening, PPE supplies, and isolation infrastructure.	Moderate/Low: Cost-effective basic hygiene supplies; easily scalable across all facility wards
Clinical Efficacy	Highly effective for outbreak control; often unsustainable or controversial in high-endemicity settings	High efficacy in endemic settings; provides broad-spectrum protection against multiple MDR threats
Patient Impact	Risk of stigmatization, psychological distress, and reduced healthcare workers contact	Integrated into routine care; lower psychological burden and improved quality of care

CRE, Carbapenem-Resistant Enterobacterales; CRAB, Carbapenem-Resistant *Acinetobacter baumannii*; MRSA, Methicillin-Resistant *Staphylococcus aureus*; PPE, Personal Protective Equipment; MDR, Multidrug-Resistant.

**Table 3:** Risk-Based Isolation Prioritization Table.

Risk Category	High Priority	Medium Priority	Low Priority
Patient Factors	Symptomatic Infection	Requirement for Intensive Medical Intervention	Asymptomatic Colonization
	Immunosuppression (SOT, HSCT)	Multi-body-site Colonization	Invasive Procedures
	Incontinence, Diarrhea	Prolonged ICU Stay	Short-term Hospitalization (<6 Weeks)
Pathogen Factors	Airborne Transmission	Moderate Transmissibility	Contact Transmission
	High Infectivity	Moderate Environmental Resilience (>1 week)	Low Infectivity
	High Environmental Resilience (>1 month) (CRAB, VRE)		Low Environmental Resilience
	Limited Treatment Options (CRE, *C. auris*)		Pathogens with High Community Prevalence
Facility Factors	Inadequate Infrastructure	Limited Infection Control Programs	Adequate Infrastructure
	Low Staff-to-Patient Rate	Insufficient Financial Support	Regularly Audited IPC Program
	Low Compliance with IPC measures		High Compliance Rate
Recommendation Action	STRICT ISOLATION	COHORTING	STANDARD PRECAUTIONS

*C. auris*: *Candidozyma auris*; CRAB: Carbapenem-Resistant *Acinetobacter baumannii*; CRE: Carbapenem-Resistant *Enterobacterales*; HSCT: Hematopoietic Stem Cell Transplantation; ICU: Intensive Care Unit; IPC: Infection Prevention and Control; SOT: Solid Organ Transplant; VRE: Vancomycin-Resistant *Enterococcus*.

**Table 4:** Criteria and Guideline Recommendations for the Discontinuation of Isolation for MDROs.

Microorganism	Standard Discontinuation Criteria	Proposed Precision/Risk-Based Strategy
ESBL-E	3 negative rectal swabs obtained at least 1 week apart during an antibiotic-free period [58]	Clinical Judgment: Routine isolation not recommended in low-risk wards Automatic removal 6 months after the last positivity
VRE	3 negative stool/rectal swabs obtained 1 week apart during an antibiotic-free period [58]	Time-Based: Termination without screening if no recurrence occurs within 3–6 months after the last positivity
MRSA	3 negative screenings (Nose, axilla, groin, wound) obtained >24 h (preferably 1 week) apart while not on systemic antibiotics [25,58]	A single negative screening may suffice for readmissions in low-prevalence settings
CRE/CRAB	Very Strict Criteria: At least 2 consecutive negative rectal swabs collected at least 1 week apart [58]	Active Follow-up: Even if isolation is discontinued, the patient should be flagged as “History of CRE” and screened at every admission
*C. auris*	NOT RECOMMENDED. Patients are considered colonized indefinitely [34]	

CRE: Carbapenem-Resistant *Enterobacterales*; CRAB: Carbapenem-Resistant *Acinetobacter baumannii*; ESBL-E: Extended-Spectrum Beta-Lactamase-producing *Enterobacterales*; MRSA: Methicillin-Resistant *Staphylococcus aureus*; MDROs: Multidrug-Resistant Organisms; VRE: Vancomycin-Resistant *Enterococcus*.

## Data Availability

Not applicable.

## Abbreviations

The following abbreviations are used in this manuscript:

BSI	Bloodstream Infection
CDC	Centers for Disease Control and Prevention
CRAB	Carbapenem-Resistant *Acinetobacter baumannii*
CRE	Carbapenem-Resistant Enterobacterales
ESBL	Extended-Spectrum Beta-Lactamase
ESBL-E	Extended-Spectrum Beta-Lactamase-producing Enterobacterales
ESCMID	European Society of Clinical Microbiology and Infectious Diseases
HSCT	Hematopoietic Stem Cell Transplantation
ICU	Intensive Care Unit
IPC	Infection Prevention and Control
MDR	Multidrug-Resistant
MDROs	Multidrug-Resistant Organisms
MRSA	Methicillin-Resistant Staphylococcus aureus
PPE	Personal Protective Equipment
R0	Basic Reproduction Number
RCT	Randomized Controlled Trial
SHEA	Society for Healthcare Epidemiology of America
SOT	Solid Organ Transplant
VRE	Vancomycin-Resistant Enterococcus
WHO	World Health Organization
